# Development and Validation of a Dried Blood Spot Assay Using UHPLC-MS/MS to Identify and Quantify 12 Antihypertensive Drugs and 4 Active Metabolites: Clinical Needs and Analytical Limitations

**DOI:** 10.1097/FTD.0000000000000984

**Published:** 2022-04-05

**Authors:** Laura E. J. Peeters, Soma Bahmany, Tim Dekker, Aya Aliawi, Bart van Domburg, Jorie Versmissen, Birgit C. P. Koch

**Affiliations:** Departments of * Department of Hospital Pharmacy and; † Department of Internal Medicine, Erasmus MC, University Medical Centre Rotterdam, Rotterdam, the Netherlands.

**Keywords:** hypertension, drug monitoring, chromatography, dried blood spot, adherence

## Abstract

As nonadherence to antihypertensive drugs (AHDs) can increase the risk of cardiovascular events, hospitalization, and higher costs, there is a need for a reliable, objective, and easy method to assess nonadherence in patients. The dried blood spot (DBS) sampling method used to measure drug concentrations meets these requirements. For detecting nonadherence, identification is more important than quantification. Owing to their use in clinical practice, it is important to measure multiple AHDs with a single method. Therefore, we developed and validated a single DBS method for 17 commonly used AHDs and 4 active metabolites using ultrahigh performance liquid chromatography–tandem mass spectrometry (UHPLC-MS/MS).

**Methods:**

Analytical validation of the DBS assay was performed in accordance with the guidelines on bioanalytical method validation of the European Medicines Agency and US Food and Drug Administration as well as the International Association of Therapeutic Drug Monitoring and Clinical Toxicology guidelines.

**Results:**

We validated 12 of the 17 AHDs according to the European Medicines Agency and Food and Drug Administration requirements for bioanalytical method validation. Eleven AHDs were validated for both identification and quantification of drug concentrations, whereas nifedipine was only validated for identification. However, 5 of the 17 AHDs were excluded due to suboptimal validation results. Lercanidipine was excluded due to nonlinearity, and all 4 AHDs measured in the negative mode of UHPLC-MS/MS were not in accordance with one or more of the acceptance criteria and were therefore excluded.

**Conclusions:**

The described method accurately measured AHDs in DBS and can be used to determine nonadherence in patients. However, method validation revealed a challenging balance between analytical limitations and clinical needs when analyzing multiple drugs using the same method.

## Abstract 2

Supplemental Digital Content is Available in the Text.

## BACKGROUND

Nonadherence to antihypertensive drugs (AHDs) is an important cause of therapeutic failure in patients with hypertension, increasing the risk of cardiovascular diseases and kidney failure.^1,2^ Identification and improvement of nonadherence is difficult; recognition by health care providers is suboptimal, and lack of this recognition often leads to overestimation of adherence.^3^ Therefore, several studies have focused on the development of new identification methods to solve this problem. One of the most objective and accurate methods to assess nonadherence to drugs is the measurement of drug concentrations in body fluids, such as blood or urine. This specific identification method is used in a research setting, but its implementation in the clinic lags behind. This is probably due to several factors that require attention before implementation, for example, the willingness of physicians to use this method and logistical problems that have to be addressed.^4–7^ Recent research studies have mainly focused on optimizing and facilitating the collection of body fluids such as blood to improve the usability of measuring drug concentrations in clinical practice.^8^ We previously presented the analytical and clinical validation of a dried blood spot (DBS) sampling method that was used to determine 8 of the most commonly used AHDs and 4 active metabolites simultaneously by means of a validated ultrahigh performance liquid chromatography–tandem mass spectrometry (UHPLC-MS/MS) method.^9,10^ With this method, we were able to accurately access nonadherence, while sampling directly at the doctor's office when nonadherence was expected. However, for multiple reasons, it is necessary to improve this method. First, our previous method included drugs from 4 different drug classes, which represent only a small part of the more than 50 available AHDs.^4,11^ Hypertension guidelines recommend the initiation of AHD treatment with 1 of 4 drug classes, including thiazide diuretics, calcium channel antagonists, angiotensin-converting enzyme inhibitors, and angiotensin II receptor blockers. The guidelines do not indicate which AHD from a drug class is preferred when no comorbidities are present. As a result, there is a large variability in drug preference among and within certain drug classes when a prescription is needed.^12,13^ Furthermore, when more than one AHD is necessary for blood pressure control, the number of drug combinations that can be applied is high. Therefore, a method to assess nonadherence to AHDs should include as many different AHDs as possible with, at least, the most commonly used AHDs. Second, most patients with uncontrolled hypertension or hypertension combined with other comorbidities, such as atrial fibrillation, use beta-blockers, which were not included in the previously validated DBS method.^11^ Therefore, a new method should include at least one beta blocker to increase its usability. Finally, accurate measurement of the lowest possible concentrations is most important when nonadherence is determined based on the drug concentration. This is necessary to prevent false inferences of nonadherence and consequently maintain an optimal patient–health care provider relationship. Based on the clinical data, we observed that the lower limit of quantification (LLOQ) and lower limit of detection (LLOD) of most of the 8 drugs determined in the previous method could be improved.^14^ Here, to improve the clinical applicability of the DBS method and increase the accuracy of measuring AHDs at low concentrations, we validated a DBS method measured with UHPLC-MS/MS that included 17 different AHDs and 4 active metabolites from 6 different drug classes.

## METHODS

The initial validation was started with 17 different AHDs and 4 of their active metabolites. We only included metabolites from AHDs, which are the so-called prodrugs. From these drugs, the parent drug is metabolized into a pharmacologically active drug. Metabolites from drugs, such as metoprolol, where the parent drug already exhibits pharmacological activity, were not included. Accurate validation of the active metabolites, in case of adherence, is most important in comparison with their associated parent drugs. The parent drugs are vastly metabolized to the active components after intake of the drug and are thereby undetectable after a few hours. This makes them less convenient to use in clinical practice.

### Chemicals and Materials

Amlodipine besylate, barnidipine, bumetanide, doxazosin, hydrochlorothiazide, irbesartan, metoprolol, nifedipine, spironolactone, telmisartan, lercanidipine, and valsartan were purchased from Sigma-Aldrich Chemie (Zwijndrecht, Netherlands). Canrenone, doxazosin-d8, enalapril, enalapril-d5, hydrochlorothiazide-13Cd2, losartan, losartan-carboxylic acid, perindopril, and perindoprilat were purchased from Santa Cruz Biotechnology (Heidelberg, Germany). Enalaprilat and candesartan were purchased from Alfa Aesar (Kandel, Germany), and chlorthalidone was purchased from EDQM (Strasbourg, France) and Cayman (Ann Arbor, MI).

Acetonitrile [99.9%, high performance liquid chromatography (HPLC)-R grade], absolute methanol [liquid chromatography/mass spectrometry (LC/MS) grade], and formic acid (FA) (99.9%, UHPLC/MS grade) were purchased from Biosolve (Valkenswaard, Netherlands). Ammonium hydroxide (AH) was purchased from Merck Millipore (Darmstadt, Germany). Deionized water was produced with a Milli-Q Advantage A10 purification system from Merck-Millipore (Darmstadt, Germany). Fresh human blood without AHDs was obtained from patients who provided consent to use the remnant material for other purposes.

### UHPLC-MS/MS Equipment and Conditions

#### Instruments

Analyses were performed using a Waters Xevo TQ-S micro UPLC-MS/MS system consisting of an Acquity Binary Solvent Manager, column manager, column aux manager, and sample manager. MassLynx V4.1 (www.waters.com) was used to analyze the data.

### Chromatographic Conditions

For chromatographic separation, an Acquity UPLC BEH reversed-phase column (2.1 × 100 mm, 1.7 µm; Waters, Milford, CT) was used. Two stages of gradient elution were performed with different mobile phases: one for the negative measurement mode and the other for the positive measurement mode. The mobile phase for the negative mode consisted of a mixture of 0.1% AH in Milli-Q water (eluent A1) or LC-MS grade methanol (B1). The mobile phase for the positive mode consisted of a mixture of 0.1% FA in Milli-Q water (eluent A2) or 0.1% FA in LC-MS grade methanol (eluent B). The same multistep gradient was used for both mobile phases with a flow of 0.3 mL/min. The program was started at an equilibrium of 95% eluent A and 5% eluent B. Over a period of 0–1.1 minutes, the concentration of eluent B was increased to 50%. From 1.1 to 2.2 minutes, eluent B was increased further to 80%, and from 2.2 to 2.3 minutes to 100%, after which a stabilization phase of 2 minutes at 100% B took place. Between 4.3 and 4.5 minutes, the gradient was set back to the original composition of 95% eluent A and 5% eluent B and equilibration from 4.5 to 6.0 minutes. The column oven was set at 70°C, and the temperature of the autosampler was at 15°C.

### Mass Spectrometry Conditions

For each compound, a solution of 1 mg/L methanol was used to optimize the MS settings. This solution was directly injected into MS without passing through a LC system. The spray voltage was set at 2.0 kV, cone voltage at 20 V, source temperature at 150°C, desolvation temperature at 350°C, cone gas flow at 10 L/h, and desolvation flow at 900 L/h. The specific MS settings for each compound are provided in **Supplemental Digital Content** (see **Table 1**, http://links.lww.com/TDM/A570).

### Standards and Control Samples

#### Preparation of Stock Solutions

Stock solutions were prepared for each drug (except for doxazosin and metoprolol) by dissolving 25 mg of the drug in 50 mL of methanol. This led to a drug concentration of 500 mg/L. For each of these latter drugs, a salt form was used, resulting in a concentration of 400 mg/L. Stock solutions of enalaprilat, losartan-CA, and perindoprilat were prepared by dissolving 10 mg of each in 25, 10, and 20 mL of methanol, leading to a concentration of 400 mg/L, 1000 mg/L, and 500 mg/L, respectively. The stock solutions were stored at −20°C away from light. The maximal storage time for the stock solutions under these conditions was 9 months after preparation.^9^ We prepared 3 different mixed solutions with these stock solutions. This was performed to minimize the retention time when reanalysis was necessary for one specific drug. The composition of these mixed solutions was based on the use of combination tablets or the availability of active metabolites. For example, amlodipine and hydrochlorothiazide are often combined with renin–angiotensin–aldosterone system inhibitors. All mixed solutions were diluted with methanol to a total volume of 25 mL. The combination of drugs per mix solution as well as the final concentrations can be found in **Supplemental Digital Content** (see **Table 2**, http://links.lww.com/TDM/A570). The first mixed solution consisted of amlodipine, candesartan, chlorthalidone, hydrochlorothiazide, irbesartan, telmisartan, perindopril, perindoprilat, and valsartan. The second mix solution consisted of barnidipine, bumetanide, canrenone, doxazosin, metoprolol, nifedipine, and spironolactone, and the third mix solution contained enalapril, enalaprilat, losartan, and losartan-CA. The mixed solutions were stored at −20°C and protected from light for a maximum time of 9 months after preparation. These storage conditions were based on stability research of stock solutions.^9^

#### Preparation of Calibration Standards and QC Samples

Calibration standards were prepared from 3 previously described mixed solutions. Three different calibration standards were prepared for each mix solution. All 3 mixed solutions were diluted 2, 4, and 25 times by adding Milli-Q. Dilutions of the calibration standard were performed by adding 50 µL to an Eppendorf tube and mixing it with 950 µL whole blood by vortexing for 10 seconds. These whole blood calibration standards were used to sample the DBS cards with a filter paper (Whatman, protein saver, 903 card). Four blood spots were sampled per standard. Cards were dried and stored in a desiccator at room temperature for at least 24 hours after sampling.

New stock and mixed solutions were prepared for quality control (QC) samples. These solutions consisted of the same groups of drugs used as calibration standards. In addition, the same amount of drug was weighed and diluted for each drug, with the exception of amlodipine and barnidipine. For the QC samples, amlodipine salt and barnidipine salt were used, which led to a final concentration of 360 mg/L amlodipine base and 465 mg/L barnidipine base. These stock solutions were added in different amounts to a mixed solution and diluted with methanol to a volume of 25 mL. The exact amount of stock solution per drug can be found in **Supplemental Digital Content** (see **Table 2**, http://links.lww.com/TDM/A570). To prepare 3 concentrations of QC samples, namely, low, medium, and high, all mixed solutions were first diluted 1.5, 5, and 21 times with Milli-Q water. Thereafter, 50 µL of all QC samples was mixed with 950 µL whole blood without the presence of AHDs, and 4 spots of 50 µL per QC were added to the DBS cards. These cards were also dried and stored in a desiccator at room temperature for at least 24 hours before use and reused during the validation process for a maximum of 2 weeks.

#### Preparation of IS Working Solution

A different internal standard (IS) was used for each stock solution. Hydrochlorothiazide-13CD2 was used as stock solution 1, doxazosin-d8 for stock solution 2, and enalapril-d5 for stock solution 3. All ISs were dissolved in methanol to a concentration of 250 mg/mL for doxazosin-d8, hydrochlorothiazide-13CD2, and 100 mg/mL for enalapril-d5. Subsequently, these 3 solutions were mixed into a final combined IS solution (100 mL) containing 20 µL doxazosin-d8 solution, 25 enalapril-d5 solution, 100 µL hydrochlorothiazide-13CD2 solution, and a 1:1 acetonitrile and methanol dilution. The stock solution of the IS was based on the stability of the previously described stock solutions and stored at −20°C protected from light for a maximum of 9 months.

### Sample Preparation

From each of the samples, a circle with a diameter of 6 mm was punched out of the DBS card. This sample was then placed in a cryotube, and 200 µL IS solution was added. Thereafter, samples were mixed by vortexing and placed in a sonication bath at 40°C for 20 minutes. Subsequently, the samples were centrifuged, and 50 µL of each sample was taken, placed in an autosampler insert vial, and 150 µL Milli-Q was added. All samples were homogenized and placed in an autosampler for analysis. The injection volume was 10 µL per sample.

### Method Development and Validation

#### Method Development and Optimization

The previous method to analyze AHDs in DBS used a UHPLC-MS/MS Thermo TSQ Vantage.^9,10^ For this new method, we switched to a UHPLC-MS/MS Waters Xevo TQ-S micro, as described earlier. This method has a higher sensitivity and, therefore, gives us the ability to decrease the LLOQ for AHDs of interest. The parameters of the mass spectrometer were optimized using the LC-MS/MS software MassLynx V4.1.

### Chromatographic Separation

Different flows were tested to determine the maximal yield of each AHD. Compared with the previous method with a flow of 0.5 mL/min, the flow was decreased to minimize dilution of the sample and a flow of 0.3 mL/min was found to be the most sensitive for analyzing all components. Candesartan, chlorthalidone, hydrochlorothiazide, and telmisartan were measured in both the negative ionization (ESI^−^) and positive ionization mode (ESI^+^). Higher peaks of these drugs were found in the negative mode; therefore, we chose to use this mode for further analysis. To further optimize the recovery of the components measured in the negative ionization mode, several eluent compositions were tested, including different gradients, acidification, and basification of the eluent. For acidification, 0.1% FA was used, whereas 0.1% and 0.5% AH were used for basification. Candesartan, chlorthalidone, hydrochlorothiazide, and telmisartan all had a higher peak surface when 0.1% AH was used. Increase in sensitivity of candesartan, chlorthalidone, and telmisartan was observed when 0.5% AH was used. Therefore, an eluent with 0.5% AH was chosen for use in the negative ionization mode. Spironolactone and canrenone have the same mass transition, which makes it difficult to determine the MS of both components. Therefore, these components were distinguished based on their retention times.

### Sample Preparation

The influence of hematocrit was tested in previous studies and showed negligible effect on the quantitative drug concentrations of 8 AHDs and 4 metabolites.^10^ These were not separately reinvestigated for newly added compounds but based on results from matrix effects.

#### Method Validation

This method was validated in accordance with the guidelines on bioanalytical method validation of the European Medicines Agency and US Food and Drug Administration combined with the International Association of Therapeutic Drug Monitoring and Clinical Toxicology guidelines for the development and validation of DBS based methods.^15–17^ For this, the following parameters were determined: linearity, LLOQ, accuracy and precision, stability, matrix effects, and carryover.

### Linearity

Based on the therapeutic range of each drug, 10 to 11 calibration standards were made from the mix standards to determine the linearity of each AHD, including the metabolites. All standards were measured in duplicates. Blank samples from whole blood, with and without IS, were measured during each run. The calibration curve was constructed by plotting the ratio of the measured drug concentration to the corresponding IS concentration against the theoretical drug concentration. In this analysis, a maximum of 2 calibration standards were discarded when the difference between the measured and theoretical concentrations was <15% or in the case of the LLOQ <20%. However, samples cannot be discarded when they are at the lowest or highest concentration or when they are consecutive. Linear regression analysis was performed to determine the coefficient r and the coefficient of determination. The coefficient had to be >0.995, and R squared had to be >0.990 for each component.

### Limits of Quantification

The LLOQ and LLOD are the most important values when nonadherence is evaluated. The theoretical LLOQ was determined by measuring blank samples spiked with IS on 6 consecutive days. Thereafter, the real LLOQ was determined by measuring a sample with the theoretical LLOQ concentrations in duplicates on 6 consecutive days. For these 3 new mix standards, the LLOQ values of the AHDs of interest were prepared. The difference between the measured and calculated concentrations is <20%. The upper limit of quantification (ULOQ) was derived from a linear experiment. In this case, accurate determination of the ULOQ is of less importance, whereas evaluating nonadherence is the mean goal.

### Accuracy and Precision

Interday and intraday accuracy and precision were determined using QC samples at low, medium, and high concentrations. For the interday accuracy and precision, all QC samples were measured in duplicate on 6 consecutive days. The intraday accuracy and precision were determined by measuring 6 replicates of each QC sample concentration. The measured concentrations were compared with the reference concentrations determined from the weighted amounts of the drug. The differences in accuracy and precision were <15%.

### Stability

In a previous study, we tested the stability of AHDs when sampled on a DBS card.^10^ However, for this method, new AHDs were added, and other combinations for the mix standards were used. Therefore, a new stability analysis is performed. QCs (low, medium, and high) of all mix standards were used in concentrations as previously described to determine the stability of the included AHDs. After sampling, the cards were dried in a desiccator for 24 hours at room temperature and protected from light before the baseline measurements were performed. The results from these first measurements were used as nominal values to determine any degradation. Residual cards were stored for 7, 14, and 28 days after sampling. Measurements were performed in duplicates. In accordance with the guidelines for the development and validation of DBS-based methods, a degradation of ±15% of the nominal value was considered acceptable.^17^

### Matrix Effects

Matrix effects were not tested separately, whereas the samples for the interday analysis were prepared with blood pooled from different patients (n = 6) and varied between the days of analysis resulting in different matrices and were measured at different concentrations (QC low, medium, and high).^17^ Therefore, data from the reproducibility analysis were used to determine any matrix effects including hematocrit. Acceptance limits for matrix effects were not stated in the guidelines for alternative sampling, but they should be as small as possible with a relative SD preferably within 15%.^17^

### Carryover

The carryover of each drug was determined in the blanks after the measurement of the highest calibration standards. The concentrations were then compared with the measured concentrations in the highest standard and represented as a percentage. The results of the blanks must be less than 20% of the LLOQ.

## RESULTS

### Analytical Validation of the Method

#### Linearity

Linearity was obtained for all analytes over the calibration range (Table 1). The correlation coefficients of all analytes were within the acceptance criteria of >0.995, except for lercanidipine and chlorthalidone. After further analysis, chlorthalidone was found to be linear with only a slightly skewed line toward the lowest QC, which was still within the preset limits (<20%). The concentrations of lercanidipine followed a nonlinear pattern at low concentrations. After careful consideration, the method was not optimized and lercanidipine was excluded from the method.

**TABLE 1.** Validation Results of DBS Assay, Including Linearity and Limits of Quantification

Compound (Metabolite)	Correlation r*	LLOD (μg/L)‡	Previous LLOD (μg/L)^9^	LLOQ (μg/L)*	Previous LLOQ (μg/L)^9^	Calibration Range (μg/L)*	Specificity (%) <25%	Carryover (%) <20%
Amlodipine	0.9960	17.1	9.00	24.1	10.9	7.8–583.2	−10.4	16.3
Barnidipine	0.9964	2.1	—	3.1	—	2.0–152.8	12.8	17.4
Bumetanide	0.9987	4.0	—	4.0	—	4.0–300.1	0.2	11.7
Candesartan (−)	0.9959	61.3	—	61.3	—	61.3–1378.8	−9.1	**20.6** †
Canrenone	0.9975	26.8	—	31.2	40.0	30.9–1543.8	2.6	0
Chlorthalidone (−)	0.9926†	0	—	24.1	—	24.1–542.7	1.4	0
Doxazosin	0.9989	18.1	—	22.0	—	16.9–1270.8	1.6	**31.1** †
Enalapril	0.9976	0.4	0.14	2.2	1.5	2.1–46.4	−12.3	**37.8** †
Enalaprilat	0.9954	1.1	4.50	2.6	10.0	2.5–37.2	−1.7	0
Hydrochlorothiazide (−)	0.9962	40.2	40.0	40.2	50.0	40.2–903.6	−17.5	**38.7** †
Irbesartan	0.9972	7.7	—	21.5	—	21.5–1932.3	0.5	5.2
Lercanidipine	0.9870†	NA	—	NA	—	1.9–71.7	NA	NA
Losartan	0.9977	1.7	5.0	4.4	5.0	4.2–190.5	−13.6	5.7
Losartan-CA	0.9982	2.6	32.0	8.7	40.0	8.4–379.1	2.2	**37.1** †
Metoprolol	0.9969	0	—	4.0	—	4.0–296.0	−2.5	0
Nifedipine	0.9950	3.5	6.7	10.1	40.0	10.0–375.8	NA	**23.3** †
Perindopril	0.9980	0.7	0.5	2.03	4.5	2.0–152.3	NA	13.6
Perindoprilat	0.9962	1.3	2.5	2.01	5.0	2.0–100.9	−19.0	9.4
Spironolactone	0.9979	5.2	18.0	10.0	20.0	7.6–381.8	2.4	0.5
Telmisartan (−)	0.9974	65.2	—	65.2	—	65.2–3259.1	−1.9	**25.9** †
Valsartan	0.9966	21.3	11.0	161.5	30.0	161.5–3633.3	−0.2	18.8

Bold denotes correlation values <0.995 and carryover >20%.

* N = 12 samples.

† Value does not meet the requirement of validation, but this is solved by lowering the ISs.

‡ Each value represents the mean of duplicate samples.

NA = not available, CA = carboxylic acid.

### Limits of Quantification

The limits of quantification and detection of all components are listed in Table 1. Both values must be as low as possible to detect nonadherence. For this purpose, the ULOQ was less important. In addition, the parent drugs are less important, while they are rapidly metabolized to their active metabolites. The LLOQ and LLOD decreased for all components as compared with the previous method, with the exception of valsartan and amlodipine.^10^ For the components that were measured in the negative mode, the LLOQ was equal to the lowest QC.

### Accuracy and Precision

The results of the interday and intraday precision and accuracy are shown in Table 2. All components measured in the positive mode were within the predetermined acceptance values at QC low, except for nifedipine. Valsartan and telmisartan displayed some interday values slightly above the accepted accuracy of 15% but were very close to this limit. The intraday accuracy from QC medium and high of bumetanide and enalaprilat both exceeded the acceptance criterion by more than 10%. Furthermore, perindoprilat was slightly above the acceptance criterion for intraday accuracy. Components measured in the negative mode showed some more deviant values, especially at low QC. The accuracy and precision of telmisartan were almost within the acceptance criteria for accuracy and precision, with an interday accuracy of <15% in QC medium.

**TABLE 2.** Validation Results of DBS Including Accuracy and Imprecision Within-Day and Between-Day

Compound	QC Concentrations (μg/L)			Within-Day (n = 6)		Between-Day (n = 12)	
		Actual	Measured*	Accuracy (Bias %)	Imprecision (CV%)	Accuracy (Bias %)	Imprecision (CV%)
Amlodipine	QC low	21.01	22.1	5.5	3.6	5.3	6.5
	QC medium	210.1	231.1	7.1	8.7	10.0	13.1
	QC high	300.1	315.4	−6.2	12.9	5.1	14.4
Barnidipine	QC low	8.2	7.9	−14.4	5.8	−2.6	9.5
	QC medium	79.0	92.4	13.6	6.0	17.1	6.0
	QC high	114.3	129.8	13.0	2.3	13.5	7.0
Bumetanide	QC low	15.5	15.4	−14.4	7.5	−0.6	8.9
	QC medium	154.7	164.5	13.6	2.6	6.3	5.0
	QC high	221.1	328.4	13.0	2.7	48.6‡	7.4
Candesartan (−)	QC low	122.7	86.8	−30.9‡	17.5	−29.2‡	21.3‡
	QC medium	563.3	553.6	−7.9	6.5	−1.7	9.2
	QC high	804.7	770.4	−17.0†	11.1	−4.3	11.1
Canrenone	QC low	77.8	72.6	−18.7†	10.0	−6.6	13.9
	QC medium	777.5	835.0	14.2	2.4	7.4	4.9
	QC high	1110.7	1189.7	12.6	2.8	7.1	6.0
Chlorthalidone (−)	QC low	41.5	34.0	−9.2	14.3	−18.2†	18.3†
	QC medium	207.7	202.8	7.6	10.8	−2.3	9.8
	QC high	296.6	294.3	7.4	11.1	−0.8	13.3
Doxazosin	QC low	67.1	60.6	−9.0	2.7	−9.7	5.4
	QC medium	671.1	681.3	3.9	3.0	1.5	5.1
	QC high	958.7	960.3	3.8	2.0	0.2	4.1
Enalapril	QC low	4.4	5.1	12.6	6.2	16.5†	6.8
	QC medium	30.6	34.2	14.2	2.3	11.8	14.5
	QC high	43.7	49.1	15.2†	9.1	12.2	5.7
Enalaprilat	QC low	3.2	3.5	16.3†	8.0	9.8	11.7
	QC medium	22.5	28.4	19.0†	3.6	26.2‡	11.2
	QC high	32.2	40.4	17.9†	13.7	25.7‡	14.1
Hydrochlorothiazide (−)	QC low	85.7	75.2	−2.6	12.4	−12.3	24.6‡
	QC medium	428.4	380.0	14.9	13.6	−11.3	10.4
	QC high	612.0	590.5	15.7†	8.8	−3.5	16.0†
Irbesartan	QC low	72.8	76.2	7.4	3.4	4.7	4.2
	QC medium	728.0	744.1	8.7	8.9	2.2	6.9
	QC high	1040.0	1067.6	10.1	7.2	2.7	9.4
Losartan	QC low	12.1	12.2	2.2	4.2	1.7	7.0
	QC medium	84.4	95.7	9.4	1.9	13.5	7.5
	QC high	120.5	135.4	12.5	8.7	12.4	5.4
Losartan-carboxylic acid	QC low	24.4	22.2	−7.8	8.4	−8.9	8.5
	QC medium	171.0	169.5	−10.1	1.9	−0.9	10.0
	QC high	244.3	239.5	−8.3	10.2	−2.0	7.7
Metoprolol	QC low	15.3	15.4	4.2	4.2	0.4	5.0
	QC medium	153.4	155.0	7.2	2.6	1.1	6.5
	QC high	219.2	220.2	3.2	3.7	0.5	6.2
Nifedipine	QC low	19.9	27.2	14.1	9.8	36.7‡	19.2
	QC medium	199.1	335.3	−9.2	3.4	68.3‡	40.8‡
	QC high	284.5	340.7	−28.0†	4.4	19.8†	27.2‡
Perindopril	QC low	7.1	4.6	−4.7	5.0	−35.7‡	27.3‡
	QC medium	71.4	42.5	−2.7	10.3	−40.5‡	30.6‡
	QC high	102.0	61.0	−0.7	9.8	−40.2‡	39.0‡
Perindoprilat	QC low	5.8	6.4	0.6	7.0	9.0	12.5
	QC medium	58.3	67.5	10.2	11.7	15.8†	3.7
	QC high	83.3	97.1	14.7	9.7	16.6†	6.7
Spironolactone	QC low	19.9	18.3	−14.2	7.5	−7.7	7.9
	QC medium	198.8	207.2	8.9	3.2	4.2	7.3
	QC high	284.0	291.3	8.0	2.2	2.6	6.3
Telmisartan (−)	QC low	280.5	266.2	16.9†	18.2†	−5.1	6.8
	QC medium	1402.2	1431.5	10.4	12.0	2.1	5.2
	QC high	2003.2	2062.8	15.0	11.5	3.0	13.1
Valsartan	QC low	140.17	153.2	19.2†	5.9	9.3	8.8
	QC medium	1401.7	1476.4	15.8†	12.5	5.3	8.7
	QC high	2002.4	2132.0	18.1†	12.8	6.5	12.7

* Each value represents the mean of duplicate samples measured.

† Value does not meet the requirement of <15%.

‡ Value does not meet the requirement of <20%.

NA, not available; CV, coefficient of variation.

### Stability

The stability of the measured drugs after sampling on a DBS card was assessed after 7, 14, and 28 days (Table 3). Most AHDs were stable at room temperature after a storage time of 28 days, apart from valsartan and irbesartan, which were stable up until 14 days after sampling. The stability of all calcium antagonists (amlodipine, barnidipine, and nifedipine) was not consistent over time with unstable samples at different QC concentrations and storage times. Therefore, a second experiment was conducted for amlodipine and barnidipine, where stability was assessed after 21 days. In this analysis, the average degradation of amlodipine after 3 weeks exceeded the limit of <15% for QC medium and high. For barnidipine, only the QC low exceeded the limit of <15%.

**TABLE 3.** Stability of 12 Antihypertensive Drugs and Their Active Metabolites; Differences Were Measured in Values at 7, 14, and 28 d

Compound [Metabolite]		Stability After 7 d (%)	Stability After 14 d (%)	Stability After 28 d (%)
Amlodipine*	QC low	NA†	NA†	5
	QC medium	NA†	NA†	−28‡
	QC high	NA†	NA†	−22‡
Barnidipine*	QC low	30‡	39‡	19‡
	QC medium	14	26‡	13
	QC high	7	11	12
Bumetanide	QC low	5	17‡	−11
	QC medium	5	5	−24‡
	QC high	10	7	−8
Canrenone	QC low	7	2	3
	QC medium	−8	2	−6
	QC high	−5	3	5
Doxazosin	QC low	0	2	−5
	QC medium	1	−6	−9
	QC high	3	4	1
Enalapril	QC low	−1	6	8
	QC medium	−2	−30†	2
	QC high	0	−1	−14
Enalaprilat	QC low	8	NA	−18‡
	QC medium	−4	−36†	−3
	QC high	−6	−11	−12
Irbesartan	QC low	−12	−18‡	−27‡
	QC medium	−10	−20‡	−29‡
	QC high	−7	−19‡	−16‡
Losartan	QC low	−2	3	−9
	QC medium	1	−26†	11
	QC high	0	1	−9
Losartan-CA	QC low	4	6	−6
	QC medium	3	−29†	4
	QC high	−4	0	−6
Metoprolol	QC low	4	7	6
	QC medium	−5	−4	−3
	QC high	0	−1	3
Nifedipine	QC low	−40‡	−50‡	−18‡
	QC medium	−4	−18	−26‡
	QC high	−5	−3	−2
Perindopril	QC low	−3	−6	−10
	QC medium	0	−9	−19‡
	QC high	0	0	−17‡
Perindoprilat	QC low	−11	6	3
	QC medium	19	−2	−5
	QC high	9	2	−2
Spironolactone	QC low	−9	−10	−19‡
	QC medium	−17‡	−12	−25‡
	QC high	−14	−10	−15
Valsartan	QC low	−8	−4	−23‡
	QC medium	−2	−3	−41‡
	QC high	−10	6	−35‡

Each value represents the mean of duplicate samples measured.

* Amlodipine stability was only analyzed after 3 weeks; barnidipine stability data after 3 weeks were added instead of 4 weeks.

† Mistake in sample preparation.

‡ > acceptance limit of 15%.

CA, carboxylic acid; NA, not available; […], active metabolite.

#### Matrix Effects and Hematocrit Value

Although the interday accuracy for all new AHDs, including doxazosin, bumetanide, barnidipine, irbesartan, and metoprolol, were within the acceptance criteria, the effects of the matrix and hematocrit were found to be nonexistent. As valsartan and enalaprilat exceeded the limits for matrix effects (>15%) at all measured concentrations, matrix effects could not be totally excluded. However, all values were <20%; therefore, the actual matrix effects will be small, which is in accordance with the guidelines.^17^

### Carryover

All components had a carryover of <20% of the LLOQ, except for candesartan, doxazosin, losartan-CA, nifedipine, and telmisartan. To decrease the carryover, the IS concentrations were lowered, as were the ULOQ of some of these components.

In Table 4, an overview is given of all 17 AHDs and 4 metabolites that were initially included in the validation and shows which ones were validated for identification and quantification or excluded from the method because the results exceeded the acceptance limits for validation.

**TABLE 4.** Overview of the Antihypertensive Drugs That Were Validated for Identification or Quantification in DBS and Those That Were Not Within the Acceptance Criteria

	Validated for Identification and Validation	Validated for Identification	Excluded from the Method
Antihypertensive drugs	Amlodipine	Nifedipine	Lercanidipine
	Barnidipine		Candesartan
	Bumetanide		Chlorthalidone
	Doxazosin		Hydrochlorothiazide
	Enalapril and enalaprilat		Telmisartan
	Irbesartan		
	Losartan and losartan-CA		
	Metoprolol		
	Perindopril and perindoprilat		
	Spironolactone and canrenone		
	Valsartan		

CA, carboxylic acid; […], active metabolite.

### Clinical Application

This method is currently used in a large multicenter trial, RHYME-RCT (Resistant HYpertension MEasure to ReaCh Targets, Dutch trial register NL6736). From this study, up to 8 samples from each newly added drug as compared with the previously validated method was selected to check the clinical applicability (see **Table 3**, **Supplemental Digital Content**, http://links.lww.com/TDM/A570). This included bumetanide, barnidipine, irbesartan, metoprolol, and doxazosin. When available, both peak and trough concentrations were selected to investigate the total analytical range. The measured concentrations of bumetanide, irbesartan, and metoprolol were all above the LLOQ. The time after intake ranged from 1.75 to 25 hours for irbesartan at a dose of 300 mg and 1.75–10 hours for metoprolol with a dose range of 12.5–100 mg. For bumetanide samples, 1.75 and 15.5 hours after intake were sampled. Only 1 of 7 samples of doxazosin concentrations was <LLOQ, but it could still be identified up to >24 hours after intake. All barnidipine concentrations were <LLOQ and were sampled up to 9.75 hours after intake.

## DISCUSSION

To identify nonadherence to AHDs in clinical practice, it is important to have a convenient and validated method to prevent false accusations. We previously published a new sampling method by means of a DBS to accurately identify and quantify 8 AHDs and 4 active metabolites from 4 different drug classes.^10^ We now present the validation of a DBS method that includes 12 AHDs and 4 active metabolites from 6 different drug classes that can be used to identify nonadherence and to quantify these drugs (with the exception of nifedipine). Our method is the first to validate this large amount of different AHDs in a single DBS. A comparable study was conducted by Kim et al,^18^ who validated a DBS method including 12 cardiovascular drugs. However, in this previous validation study, no real blood samples were used but analytical samples were diluted in sodium chloride 0.9%. Furthermore, active metabolites of AHDs were not included, and for amlodipine, the accuracy and precision exceeded the acceptance criteria. By contrast, we also found some deviating values for accuracy and precision but only for perindopril and nifedipine. These findings suggest that DBS is a difficult sampling method to simultaneously validate multiple drugs. However, this limitation is inherently coupled to the sampling process, which is more prone to variations between samples as compared with plasma samples.^19^ Punt et al^4^ included multiple AHDs in a single HPLC-MS/MS method. They developed a method that determined 52 drugs in plasma that are used in cardiovascular diseases.^4^ A major limitation of this method was the use of population trough concentrations instead of the LLOD. In previous research, we already showed that there is a large interpatient variability in AHD concentrations, also at trough concentrations, which makes the LLOD the most important value to assess adherence.^20^ This was also confirmed by a meta-analysis by Groenland et al,^21^ who warned for the use of pooled trough concentrations when assessing nonadherence. Furthermore, the study by Punt et al^4^ did not determine how long after intake they could measure drug concentrations. As degradation of AHDs in blood is relatively fast, mostly within 24 hours after intake, this is important to take into account. Finally, the stability of the 12 compounds in plasma at room temperature was less than 20% after 12 hours of storage, which makes it more difficult to use in clinical practice. The stability of most AHDs in DBS in our validation was assessed for a longer period and showed that most drugs were stable up to 21 or 28 days after sampling. In addition, none of the AHDs were below the detection limit after storage for 28 days at room temperature. However, more stability research should be performed on the currently used stock solutions, mix standards, and IS mixtures to confirm storage times and possibly increase the accuracy of our current method. In comparison with our previous validation study, we were able to lower the LLOQ as well as the LLOD for 6 of the 8 AHDs, including losartan, enalapril, perindopril, nifedipine, hydrochlorothiazide, and spironolactone. Because of this improvement in LLOQ for most AHDs, the LLOQ of amlodipine and valsartan increased slightly in our new method. We previously showed that this increase was not of clinical relevance, whereas the valsartan concentrations measured in a clinical validation study never exceeded the LLOD at 24 hours after intake.^20^ For amlodipine, we previously observed that concentrations in DBS are usually higher than those in plasma.^10^ A higher LLOQ and LLOD would therefore not directly result in false negative results measured in DBS. Because of this, we chose to exclude this minor increase in LLOQ for valsartan and amlodipine, while the LLOQ of the other drugs improved. To further improve the LLOQ, we recommend using a larger diameter for the punch, for instance, a diameter of 8 mm instead of 6 mm.^22,23^ In particular, for DBS methods where multiple drugs are analyzed, a larger punch diameter will result in a larger sample and thereby increase the amount of drug that can be measured. This, in turn, could result in a smaller LLOQ for all the measured drugs. We also found some problems with the carryover of some compounds that exceeded the acceptance limits. However, in clinical practice, we do not expect any problems with carryover, while the highest concentrations used in this validation almost never occur in clinical practice and are not clinically relevant because the main aim is to assess adherence. Furthermore, for the 3 compounds, this problem could be linked to the use of IS. Therefore, the concentration of the ISs was lowered to prevent any future carryover problems. The therapeutic ranges of the newly added AHDs were based on plasma concentrations. For some drugs, such as amlodipine, drug concentrations and the corresponding LLOQ were higher in DBS than in the plasma^10^; however, it remains unclear if the LLOQ values for barnidipine, metoprolol, irbesartan, doxazosin, and bumetanide are low. The first clinical data showed no undetectable concentrations of irbesartan, doxazosin, and metoprolol, and for bumetanide, the results seem promising. Furthermore, in a rare case in which a patient has an ultrarapid phenotype of a CYP enzyme responsible for rapid metabolism of an AHD, we are able to recognize this by measuring drug concentrations combined with information on the intake of the drug.^24^ This makes further improvement of the LLOQ less important. However, these first clinical data also showed that the barnidipine concentrations in real patients are lower than expected and lowering the current LLOD could be necessary.^4,25^ Finally, it should be mentioned that our DBS method can also be used to quantify 11 AHDs, which can contribute to the optimization of AHD treatment. The limitation of this study lies in the problem of accurately validating all AHDs in accordance with current guidelines. First, lercanidipine was found to have nonlinear properties at low concentrations, which made accurate determination at trough concentrations difficult. Therefore, to save time, the method for this compound was not further developed. In addition, all components measured in the negative mode were not in accordance with one or more of the acceptance criteria. Although we were able to measure all the components, we could not determine these values with sufficient certainty. Further optimization is possible, but it is time consuming and results in 2 separate methods for analyzing 1 sample. Furthermore, for drugs such as hydrochlorothiazide and chlorthalidone, elimination of this mode has minimal impact on the determination of nonadherence in clinical practice because these drugs are often used in combination tablets. Finally, nifedipine was the only AHD for which identification was possible, but correct quantification could not be established. However, for the purpose of measuring nonadherence, identification is more critical than quantification.

## CONCLUSIONS

The described method accommodates accurate measurements of AHDs in DBS. This method can not only be used to determine nonadherence in patients but also to provide reliable quantitative values that can contribute to personalized treatment. The use of DBS assures quick and easy sampling and is therefore an ideal strategy to use in clinical practice to assess nonadherence to AHDs. However, this validation revealed a challenging balance between the analytical limitations and clinical needs.

## Supplementary Material

SUPPLEMENTARY MATERIAL
