# Supplementary material for: Development and Validation of a Dried Blood Spot Assay Using UHPLC-MS/MS to Identify and Quantify 12 Antihypertensive Drugs and 4 Active Metabolites: Clinical Needs and Analytical Limitations
Source: Ther Drug Monit. 2022 Apr 5;44(4):568–77. doi: 10.1097/FTD.0000000000000984 (PMC9275854; doi:10.1097/FTD.0000000000000984)
Supplement: SUPPLEMENTARY MATERIAL [file tdm-44-568-s001.docx]

**Supplemental Material**

**Supplementary Table 1. Tandem mass spectrometry (MS/MS) settings.**

| **Compound**  **[metabolite]** | **Parent mass m/z** | **Daughter mass m/z** | **Dwell time (ms)** | **Cone voltage (V)** | **Collision energy (V)** | **ESI mode** |
| --- | --- | --- | --- | --- | --- | --- |
| **Amlodipine** | 409.2 | 238.1 | 10 | 10 | 8 | + |
| **Barnidipine** | 492.2 | 91.0 | 10 | 10 | 50 | + |
| **Bumetanide** | 365.2 | 240.1 | 10 | 10 | 18 | + |
| **Candesartan** | 439.0 | 309.1 | 10 | 10 | 22 | - |
| **[Canrenone]** | 341.5 | 107.1 | 10 | 10 | 60 | + |
| **Chlorthalidone** | 336.9 | 146.0 | 10 | 10 | 18 | - |
| **Doxazosin** | 452.2 | 344.2 | 10 | 10 | 30 | + |
| **Doxazosin-d8** | 460.2 | 352.2 | 10 | 10 | 34 | + |
| **Enalapril** | 377.3 | 234.2 | 10 | 10 | 18 | + |
| **[Enalaprilat]** | 349.2 | 206.1 | 10 | 10 | 18 | + |
| **Enalapril-d5** | 382.2 | 239.1 | 10 | 26 | 20 | + |
| **HCTZ (-)** | 295.9 | 268.9 | 10 | 10 | 18 | - |
| **HCTZ-13C,d2** | 300.9 | 272.0 | 10 | 10 | 18 | - |
| **Irbesartan** | 429.2 | 207.1 | 10 | 10 | 28 | + |
| **Lercanidipine** | 612.4 | 100.1 | 10 | 10 | 42 | + |
| **Losartan** | 423.2 | 207.1 | 10 | 10 | 22 | + |
| **[Losartan-CA]** | 437.1 | 235.0 | 10 | 10 | 16 | + |
| **Nifedipine** | 347.1 | 253.8 | 10 | 6 | 22 | + |
| **Metoprolol** | 268.2 | 116.1 | 10 | 10 | 18 | + |
| **Perindopril** | 369.3 | 172.2 | 10 | 10 | 22 | + |
| **[Perindoprilat]** | 341.2 | 98.0 | 10 | 10 | 34 | + |
| **Spironolactone** | 341.3 | 107.1 | 10 | 10 | 34 | + |
| **Telmisartan** | 515.1 | 267.2 | 10 | 10 | 52 | - |
| **Valsartan** | 436.3 | 235.1 | 10 | 10 | 18 | + |

*HCTZ = hydrochlorothiazide, CA = carboxylic acid*

**Supplementary Table 2. Concentrations of stock and mix solutions for the validation of antihypertensive drugs sampled with a dried blood spot (DBS).**

| **Compound**  **[metabolite]** | **Concentration**  **Stock solution**  **(mg/L)** | **Necessary volume stock solution (µL)** | **Drugs combined in mix solutions (mix 1, 2, or 3)** | **Concentration in mix solutions (µg/L)** | **Concentration mix solution**  **(µg/L) (ca.)** | | | **Concentration**  **DBS standard**  **(µg/L) (ca.)** | | |
| --- | --- | --- | --- | --- | --- | --- | --- | --- | --- | --- |
|  |  |  |  |  | **1** | **2** | **3** | **1** | **2** | **3** |
| **Amlodipine** | 500.0 | 750.0 | 1 | 15000 | 30 | 120 | 375 | 0.9 | 9.0 | 27 |
| **Barnidipine** | 500.0 | 2000.0 | 2 | 40000 | 40 | 400 | 1200 | 2.0 | 20 | 60 |
| **Bumetanide** | 500.2 | 1.0 | 2 | 10000 | 100 | 1000 | 3000 | 5.0 | 50 | 150 |
| **Candesartan (-)** | 510.6 | 1.0 | 1 | 6000 | 60 | 600 | 1800 | 3.0 | 30 | 90 |
| **[Canrenone]** | 514.6 | 1.0 | 2 | 40000 | 400 | 4000 | 12000 | 20 | 200 | 600 |
| **Chlorthalidone (-)** | 502.6 | 1.0 | 1 | 2000 | 20 | 200 | 600 | 1.0 | 10 | 30 |
| **Doxazosine** | 400.0 | 1.0 | 2 | 32000 | 320 | 3200 | 9600 | 16 | 160 | 480 |
| **Doxazosine-d8** | 250.0 | 1.0 | - | - | - | - | - | - | - | - |
| **Enalapril** | 515.8 | 1.0 | 3 | 2500 | 25 | 250 | 750 | 1.25 | 12.5 | 37.5 |
| **[Enalaprilat]** | 412.8 | 1.0 | 3 | 2000 | 20 | 200 | 600 | 1.0 | 10 | 30 |
| **Enalapril-d5** | 100.0 | 1.0 | - | - | - | - | - | - | - | - |
| **HCTZ (-)** | 510.2 | 1.0 | 1 | 10000 | 100 | 1000 | 3000 | 5.0 | 50 | 150 |
| **HCTZ-13C,d2** | 250.0 | 1.0 | - | - | - | - | - | - | - | - |
| **Irbesartan** | 536.8 | 1.0 | 1 | 200 | 2 | 20 | 60 | 0.1 | 1.0 | 3 |
| **Lercanidipine** | 506.5 | 1.0 | - | 1000 | 10 | 100 | 300 | 0.5 | 5.0 | 15 |
| **Losartan** | 529.2 | 1.0 | 3 | 16000 | 160 | 1600 | 4800 | 8.0 | 80 | 240 |
| **[Losartan-CA]** | 1053.0 | 1.0 | 3 | 16000 | 160 | 1600 | 4800 | 8.0 | 80 | 240 |
| **Metoprolol** | 505.4 | 1.0 | 2 | 10000 | 100 | 1000 | 3000 | 5.0 | 50 | 150 |
| **Nifedipine** | 501.2 | 1.0 | 2 | 2500 | 25 | 250 | 750 | 1.25 | 12.5 | 37.5 |
| **Perindopril** | 508.0 | 1.0 | 1 | 2000 | 20 | 200 | 600 | 1.0 | 10 | 30 |
| **[Perindoprilat]** | 447.5 | 1.0 | 1 | 1000 | 10 | 100 | 300 | 0.5 | 5.0 | 15 |
| **Spironolactone** | 509.0 | 1.0 | 2 | 1600 | 16 | 160 | 480 | 0.8 | 8.0 | 24 |
| **Telmisartan (-)** | 543.2 | 1.0 | 1 | 30000 | 300 | 3000 | 9000 | 15 | 150 | 450 |
| **Valsartan** | 504.6 | 1.0 | 1 | 80000 | 800 | 8000 | 24000 | 40 | 400 | 1200 |

*DBS = dried blood spot, HCTZ = hydrochlorothiazide, CA = carboxylic acid*

| **Drug** | **Half-life (h)** | **Plasma drug concentration 24-h after intake (µg/L)*** | **Measured concentrations in whole blood (DBS) (µg/L)**** | **Time between intake and sampling (h)** | **Drug dose (mg)** |
| --- | --- | --- | --- | --- | --- |
| **Doxazosin** | 10–50^1^ | 20 µg/L (dose 8 mg, at steady state)^1^ | 24.46 | 4 | 8 |
|  |  |  | 51.00 | 17 | 8 |
|  |  |  | 50.08 | 2.5 | 8 |
|  |  |  | 68.37 | 1.75 | 8 |
|  |  |  | 42.35 | 21 | 8 |
|  |  |  | 16.75 | 26.75 | 8 |
|  |  |  | 37.17 | 14 | 8 |
| **Bumetanide** | 1^2^ | 10 µg/L (dose 3 mg, 5 h after intake)^3^ | 23.42 | 2.5 | 1 |
|  |  |  | 68.37 | 1.75 | 0.5 (twice daily) |
|  |  |  | 48.17 | 15.5 | 5 (twice daily) |
|  |  |  | 59.47 | 4 | 5 (twice daily) |
|  |  |  | 27.88 | 4 | 1 |
| **Barnidipine** | 20^4^ | <2 µg/L (dose 20 mg)^5^ | 2.10 | 6.75 | 20 |
|  |  |  | 1.30 | 2.25 | 20 |
|  |  |  | 1.85 | 9.75 | 20 |
|  |  |  | 1.30 | 2.5 | 20 |
|  |  |  | 1.75 | 1.96 | 20 |
|  |  |  | 1.37 | 3.5 | 20 |
| **Irbesartan** | 11^6^ | 500 µg/L (dose 300 mg)^6^ | 12.47 | 3 | 300 |
|  |  |  | 785.45 | 2 | 300 |
|  |  |  | 89.48 | 2.25 | 300 |
|  |  |  | 729.96 | 24 | 300 |
|  |  |  | 439.76 | 25 | 300 |
| **Metoprolol (sustained release tablets)** | 3.5^7,8^ | 50 µg/L (dose 100 mg)^7^ | 87.89 | 3.3 | 100 |
|  |  |  | 10.22 | 2.25 | 50 |
|  |  |  | 5.44 | 2.25 | 12.5 |
|  |  |  | 29.67 | 1.75 | 100 |
|  |  |  | 47.81 | 7.75 | 50 |
|  |  |  | 31.54 | 5.25 | 100 |
|  |  |  | 28.43 | 5 | 100 |
|  |  |  | 11.30 | 10 | 50 |

**Supplementary Table 3. Overview of the measured concentrations of drugs, including doxazosin. bumetanide, barnidipine, irbesartan and metoprolol in whole blood sampled with a dried blood spot.**

** No clinical validation was performed, so the ratios of drug concentrations in the plasma and whole blood are unknown.
** Data were retrieved from the RHYME-RCT (*Resistant HYpertension MEasure to ReaCh Targets, Dutch trial register NL6736) *trial to determine non-adherence.
DBS = dried blood spot*

**References**

1. Chung M, Vashi V, Puente J, et al. Clinical pharmacokinetics of doxazosin in a controlled-release gastrointestinal therapeutic system (GITS) formulation. *Br J Clin Pharmacol.* 1999;48(5):678-687.

2. Holazo AA, Colburn WA, Gustafson JH, et al. Pharmacokinetics of bumetanide following intravenous, intramuscular, and oral administrations to normal subjects. *J Pharm Sci.* 1984;73(8):1108-1113.

3. Cook JA, Smith DE, Cornish LA, et al. Kinetics, dynamics, and bioavailability of bumetanide in healthy subjects and patients with congestive heart failure. *Clin Pharmacol Ther.* 1988;44(5):487-500.

4. Malhotra HS, Plosker GL. Barnidipine. *Drugs.* 2001;61(7):989-996.

5. Teramura T, Watanabe T, Higuchi S, et al. Pharmacokinetics of barnidipine hydrochloride, a new dihydropyridine calcium channel blocker, in the rat, dog and human. *Xenobiotica.* 1995;25(11):1237-1246.

6. Marino MR, Langenbacher K, Ford NF, et al. Pharmacokinetics and pharmacodynamics of irbesartan in healthy subjects. *J Clin Pharmacol.* 1998;38(3):246-255.

7. Stout SM, Nielsen J, Welage LS, et al. Influence of metoprolol dosage release formulation on the pharmacokinetic drug interaction with paroxetine. *J Clin Pharmacol.* 2011;51(3):389-396.

8. Punt AM, Stienstra NA, van Kleef MEA, et al. Screening of cardiovascular agents in plasma with LC-MS/MS: A valuable tool for objective drug adherence assessment. *J Chromatogr B Analyt Technol Biomed Life Sci.* 2019;1121:103-110.
